# Large-Scale Integration of DICOM Metadata into HL7-FHIR for Medical Research

**DOI:** 10.1055/a-2521-4250

**Published:** 2025-04-15

**Authors:** Alexa Iancu, Johannes Bauer, Matthias S. May, Hans-Ulrich Prokosch, Arnd Dörfler, Michael Uder, Lorenz A. Kapsner

**Affiliations:** 1 Friedrich-Alexander-Universität Erlangen-Nürnberg, Medical Informatics, Erlangen, Germany; 2 Bavarian Cancer Research Center, Erlangen, Bayern, Germany; 3 Medical Center for Information and Communication Technology, Universitätsklinikum Erlangen, Erlangen, Bayern, Germany; 4 Institute of Radiology, Universitätsklinikum Erlangen, Erlangen, Bayern, Germany; 5 Department of Neuroradiology, Universitätsklinikum Erlangen, Erlangen, Bayern, Germany

**Keywords:** radiology information system, health information interoperability, medical informatics applications, radiology department, hospital

## Abstract

**Background** The current gap between the availability of routine imaging data and its provisioning for medical research hinders the utilization of radiological information for secondary purposes. To address this, the German Medical Informatics Initiative (MII) has established frameworks for harmonizing and integrating clinical data across institutions, including the integration of imaging data into research repositories, which can be expanded to routine imaging data.

**Objectives** This project aims to address this gap by developing a large-scale data processing pipeline to extract, convert, and pseudonymize DICOM (Digital Imaging and Communications in Medicine) metadata into “ImagingStudy” Fast Healthcare Interoperability Resources (FHIR) and integrate them into research repositories for secondary use.

**Methods** The data processing pipeline was developed, implemented, and tested at the Data Integration Center of the University Hospital Erlangen. It leverages existing open-source solutions and integrates seamlessly into the hospital's research IT infrastructure. The pipeline automates the extraction, conversion, and pseudonymization processes, ensuring compliance with both local and MII data protection standards. A large-scale evaluation was conducted using the imaging studies acquired by two departments at University Hospital Erlangen within 1 year. Attributes such as modality, examined body region, laterality, and the number of series and instances were analyzed to assess the quality and availability of the metadata.

**Results** Once established, the pipeline processed a substantial dataset comprising over 150,000 DICOM studies within an operational period of 26 days. Data analysis revealed significant heterogeneity and incompleteness in certain attributes, particularly the DICOM tag “Body Part Examined.” Despite these challenges, the pipeline successfully generated valid and standardized FHIR, providing a robust basis for future research.

**Conclusion** We demonstrated the setup and test of a large-scale end-to-end data processing pipeline that transforms DICOM imaging metadata directly from clinical routine into the Health Level 7-FHIR format, pseudonymizes the resources, and stores them in an FHIR server. We showcased that the derived FHIRs offer numerous research opportunities, for example, feasibility assessments within Bavarian and Germany-wide research infrastructures. Insights from this study highlight the need to extend the “ImagingStudy” FHIR with additional attributes and refine their use within the German MII.

## Introduction

The essential role of medical imaging in clinical diagnostics, therapy, and disease documentation is immense and expected to continue growing, driving the development of new evaluation methods to further optimize the standard of care. 1 These data are crucial for patient-centered, personalized health care and drive the development of new evaluation methods to further optimize treatment standards.

The digitalization of health care has transformed the management and exchange of medical data. A milestone in this process was the introduction of the Digital Imaging and Communications in Medicine (DICOM) 2 standard in 1992. DICOM not only defines an interoperable data format for storing medical images but also includes metadata encapsulated in structured tags (e.g., *PatientID* , *StudyInstanceUID* ). 3 This ensures compatibility across different systems and forms the backbone of digital imaging workflows in modern health care. Additionally, Picture Archiving and Communication Systems (PACS) have become indispensable for managing, storing, and retrieving imaging data efficiently. Seamless integration of imaging data and metadata with other clinical information, such as diagnoses, procedures, and laboratory data, is essential to achieve comprehensive insights, especially with regard to secondary use. 4 Structured preparation and provision of medical image data and metadata for research purposes pose a challenge but offer the potential for significant advancements in personalized medicine and scientific discoveries. In recent years, the Health Level 7 (HL7) Fast Healthcare Interoperability Resources (FHIR) 5 standard has emerged as a robust framework for health care data exchange. FHIR organizes data into modular resources, such as *Patient* , *Observation* , and *Medication* . Of particular relevance to this project is the *ImagingStudy* resource, designed specifically to represent DICOM imaging metadata and their associated metadata. By adopting this standard, imaging data can be integrated seamlessly into broader clinical data repositories, enabling their reuse in research and clinical decision-making.

The project was pursued at the University Hospital Erlangen (UHE) in close cooperation between the Radiology Department and the local Data Integration Center (DIC). The DIC established at UHE as part of the German Medical Informatics Initiative (MII), 6 serves as a hub for harmonizing and integrating clinical data from heterogeneous departmental systems. The MII, a nationwide effort, aims to improve the reuse of medical data for research and patient care. Within this framework, the MIRACUM consortium 7 has provided key concepts and infrastructure to support the establishment of DICs. According to the Germany-wide consented definition of the MII core dataset, the DICs' integrated research data repositories are built on an FHIR server and data are provided as FHIR. 8 At UHE, this repository includes, for example, patient demographics, encounters, diagnoses, laboratory results, medication, and intensive care unit data. As of December 2023, this project marked the first effort to extend the repository by including information on imaging data. By leveraging the *ImagingStudy* FHIR type, this integration represents a step toward enabling comprehensive, multimodal research datasets that combine imaging data with other clinical parameters.

Routine imaging data from two departments of the UHE provided the foundation for developing a data processing pipeline and for conducting a comprehensive analysis of the generated FHIR output. These efforts aim to evaluate the quality and availability of DICOM metadata in those resources for secondary use in research, ultimately facilitating more effective and targeted studies.

## Objectives

The primary objective of this work was to establish an end-to-end data processing pipeline at UHE that converts DICOM metadata into the HL7 FHIR format, addressing the current gap in the integration of imaging data into research repositories. Key requirements for the pipeline included full automation of the processing once initiated, pseudonymization of all identifying attributes, mapping of values to standard terminologies, ensuring valid FHIR outputs, and seamless integration into the DIC infrastructure. Additionally, the study aimed to analyze the FHIR output generated by the pipeline to assess the quality and availability of the DICOM metadata in those generated resources, to evaluate their suitability for research applications such as feasibility queries, and to identify areas for future improvements.

## Methods

### Materials

The extraction of DICOM data from the routine PACS was built on Python-based in-house scripts developed by the local DIC team, which were adapted to address the requirements of the established pipeline. The PACS system used at UHE is synedra AIM, 9 which supports a web interface to query imaging studies and send them to DICOM endpoints. Basically, the Python script processes a list of accession numbers (identifiers) and sends the corresponding studies to a defined endpoint.

The developed pipeline uses the Radiological Society of North America (RSNA) MIRC CTP (Medical Imaging Resource Center Clinical Trial Processor) 10 11 tool as DICOM endpoint. This software framework is designed to manage and process medical imaging data for clinical trials. It provides functionalities for handling DICOM studies, including sending, storing, and pseudonymizing imaging data to ensure compliance with research privacy standards. As there was previous experience with this tool at the DIC at UHE, it was a natural choice using the CTP tool also for this project. For the DICOM-to-FHIR pipeline, a new CTP instance was parametrized as a DICOM endpoint only, with pseudonymization components disabled, to receive and store DICOM files for further processing.

The subsequent DICOM-to-FHIR conversion builds upon the open-source tool “dicom-fhir-converter” published by LinuxForHealth on GitHub, 12 which converts DICOM metadata of individual studies into FHIR. This tool was adapted and enhanced throughout this work, e.g., with the mapping of body regions to SNOMED-CT terminology. 13

To further process the generated FHIR data, we used Apache Kafka 14 as a messaging and data streaming platform within the pipeline, as it is state-of-the-art at the DIC at UHE. Apache Kafka utilizes topics to organize the flow of data and is used in the pipeline to store the converted FHIR in a dedicated topic.

Finally, the resources were pseudonymized using the existing FHIR pseudonymizer from the local DIC and loaded into an FHIR server. To connect all the pipeline components in a sequential order and to trigger steps conditionally based on the state of previous steps, we developed an application programmer interface (API) that is publicly available on GitHub, 15 to orchestrate the entire DICOM-to-FHIR pipeline, enabling the seamless transfer, and processing of DICOM data. It efficiently manages the sequential flow of data, including the conversion, storage and deletion of incoming DICOM studies in a continuous cycle. The developed API is based on Python Flask. 16

Kubernetes 17 was used to manage the deployment and scaling of the pipeline's services. It provides a robust environment for containerized applications, ensuring high availability and easy scalability across the different stages of the pipeline. Both the CTP tool with the DICOM endpoint and the self-developed API are hosted in the local Kubernetes cluster. The quality and availability of the DICOM metadata in the generated FHIR resources was evaluated using Pathling 18 and Apache Spark. 19 A Python script was developed to query data from the pseudonymized Kafka topic and to aggregate parameters using Spark DataFrames. The final results are then exported as CSV files. The Pathling framework simplifies working with FHIR data, whereas Spark ensures efficient processing of large datasets.

### Generation of ImagingStudy FHIR resources

The DICOM-FHIR converter 11 generates an “ImagingStudy” FHIR resource, following the HL7-FHIR core specification and reflecting the hierarchical structure of the original DICOM study. We modified the existing converter by changing the output type from a single resource to a FHIR bundle, which is necessary to use Kafka as a stream processor.

The DICOM-FHIR converter scripts were further refined to comply with newer versions of dependencies and to enhance compliance with DIC standards, including optimizations to the subject attribute for patient ID alignment. Additionally, the converter was enhanced to map the DICOM tag “Body Part Examined” (0018,0015) to SNOMED-CT codes, using an existing mapping table published in the DICOM standard. 20 The changes that were made to the pre-existing converter are available on GitHub. 12

### Analysis of the Pipeline Performance and the Generated FHIR data

For a large-scale test of the developed pipeline, a total of 157,379 eligible DICOM studies from the two UHE departments “Institute of Diagnostic Radiology” and “Neuroradiology” that were acquired within the year 2022 were identified by their accession numbers and sent from the PACS to the dedicated DICOM endpoint. To evaluate the total transmission time, logging files were assessed to identify the time stamp of the first DICOM file received at the CTP DICOM endpoint and the time point when the last FHIR resource has been generated. The delta between those time points was then computed and served as the measurement of the total transmission and conversion time. Also, the percentage of successfully transmitted and converted studies was collected. Furthermore, logging files were assessed for common errors and to measure the pipeline's total and average processing times.

The generated “ImagingStudy” resources were analyzed using a Python script, utilizing Pathling 18 and Apache Spark 19 to assess the quality and availability of the DICOM metadata.

The predefined analysis parameters included the number of DICOM studies and series, series grouped by modality and body region, field completion rates for laterality, body region, and modality, and the average number of series and instances per study.

### Ethical Considerations

The authors declare that this research was performed in compliance with the World Medical Association Declaration of Helsinki on Ethical Principles for Medical Research Involving Human Subjects.

This technical proof-of-concept study focused on the development and feasibility and performance evaluation of a data processing pipeline within the research data infrastructure (DIC) at the UHE. Approval from the local data protection officer was obtained for this study; however, since no clinical research study was conducted, formal ethics approval was not necessary.

## Results

### Setting up the Imaging Data Processing Pipeline

**Fig. 1:**
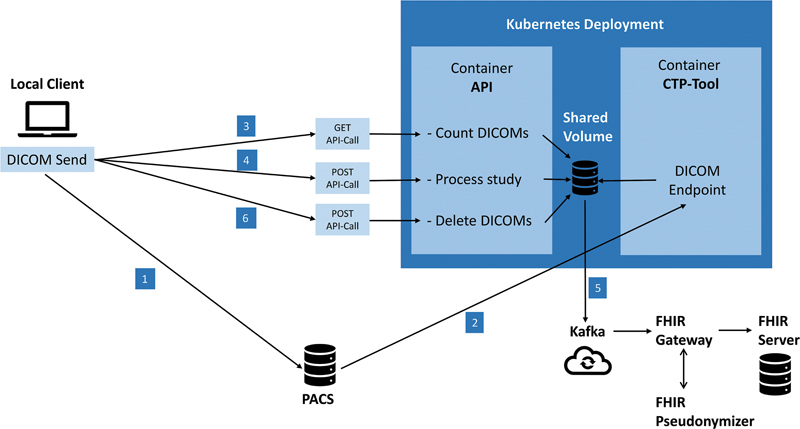
Structure of the developed DICOM-to-FHIR pipeline including the order of the individual steps: Triggering transmission from the PACS, API query, conversion to HL7-FHIR format, integration into the local FHIR gateway, pseudonymization, integration into the FHIR server. API, application programmer interface; DICOM, Digital Imaging and Communications in Medicine; FHIR, Fast Healthcare Interoperability Resources; HL7, Health Level 7; PACS, Picture Archiving and Communication Systems.

The synedra AIM 9 PACS system that is used at UHE offers a Representational State Transfer application programming interface (REST API) for querying, filtering, and transmitting DICOM studies. Unfortunately, this REST-API does not support to only extract DICOM header data, thus requiring us to extract complete DICOM files including pixel data from the PACS. Hence, the pipeline's first component comprises the sending of DICOM studies from the local PACS to a DICOM endpoint at which further processing is performed (see Fig. 1 steps 1–2). This first step was built upon the RSNA CTP. 10

An automated data processing pipeline requires reliable, sequential DICOM study processing. Neither the UHE PACS nor the CTP DICOM endpoint were able to provide information on when a DICOM study has entirely successfully been received at the DICOM endpoint; thus, a custom interface was developed using Python Flask. 16 The API interface allows real-time monitoring of incoming DICOM objects in the import folder, supports POST requests for initiating DICOM-to-FHIR conversion, and finally cleaning up DICOM objects before the processing of the next study. The pipeline processes each DICOM study sequentially as follows:

1. Send the DICOM study using its accession number via REST API call from the PACS to the CTP DICOM endpoint (see Fig. 1 steps 1–2).
2. Count the incoming DICOM objects (see Fig. 1 step 3).
3. Once all expected objects are present at the CTP DICOM endpoint, start the generation of the “ImagingStudy” FHIR resource (see Fig. 1 step 4).
4. Load resource to Kafka stream with subsequent pseudonymization and storage in FHIR server (see Fig. 1 step 5).
5. Delete all DICOM objects in the CTP DICOM endpoint (see Fig. 1 step 6).

This method prevents file mixing, maintains the required folder structure, and addresses storage issues, as retaining all studies would require approximately 100 terabytes at UHE annually.

To ensure modularity and flexibility, the setup was deployed at the DIC-internal Kubernetes 17 cluster. The pipeline consists of two containers—one for our custom REST API and one for the CTP DICOM endpoint—sharing a persistent K8s-internal storage volume.

The pipeline integrates with the DIC FHIR Gateway 21 for pseudonymizing FHIR using Apache Kafka 14 for real-time data transmission. Each generated FHIR resource is encapsulated in a bundle for efficient Kafka processing. The pseudonymization process ensures that no identifiable data remains, with cryptographic hashing applied to DICOM UIDs and pseudonymization of accession numbers and Patient IDs managed by a trustee.

The pseudonymized FHIR bundles are then available for research purposes and can be loaded onto an FHIR server, ensuring secure and standardized data handling compliant with DIC standards.

### Pipeline Performance Evaluation

After establishing the pipeline as described in the previous subsection, it was initially tested using mock data to ensure functionality and reliability before proceeding to the evaluation phase with actual data. Throughout the evaluation phase, the pipeline was able to successfully process a total of 151,090 DICOM studies over a period of around 26 days, leading to an average processing rate of 4.2 studies per minute and a completion rate of around 96%. The average size was 115 DICOM objects per study. The most common reason for a failed transfer was a faulty transfer from the PACS system due to a difference of the expected number (as requested via the PACS's REST API) and the actual number of incoming DICOM objects, which was counted by our self-developed API. This error was mainly attributable to the fact that for technical reasons, the CTP DICOM endpoint was unable to accept certain “Service Object Pairs” Instance UIDs.

### Results of the Data Analysis

As expected, the data analysis highlighted a consistent presence of the DICOM tag “ *Modality* ” ( *0008,0060* ) across the dataset. Most DICOM series, approximately 93%, were generated by so-called “ *Acquisition Modalities* ,” with computed tomography (CT) and magnetic resonance imaging comprising the majority, followed by digital radiography and angiography. “ *Non-acquisition modalities* ,” in particular structured reporting (SR), accounted for approximately 7% of the series.

The analysis of the DICOM tag “ *Body Part Examined* ” revealed that a significant portion of DICOM series lacked information about the examined body region (32%). This arises partly from non-acquisition series, where no body region is expected, but still 24% of image-acquisition series remained with missing specification of the body region examined. The most frequent values for the body regions examined were: head (19%), thorax (14%), brain (9%), and heart (8%). Moreover, the distribution of examined body regions varied across different modalities, reflecting the specialties of the clinics and the imaging techniques used, i.e., magnetic resonance scans emphasizing soft tissues like the brain, heart, and chest, whereas CT scans focus on bony structures such as the head and dense tissues like lungs and abdomen. Notably, angiography series had no entries for examined body regions, primarily due to system limitations.

The DICOM tag “ *Laterality* ” ( *0020,0060* ) is utilized to specify the lateral orientation of anatomical objects within images. Despite standardized coding for lateral positions, our comprehensive analysis found that the majority of the analyzed series (95%) lacked valid values for laterality. As expected, many series with laterality values depicted bilateral anatomical structures, with frequencies of left and right sides relatively balanced. However, the code indicating unpaired structures (“U”) was seldom used, possibly due to redundancy. The most frequently examined body regions using laterality values were identified, revealing knees and shoulders as the top regions, followed by feet, ankles, and hands. Interestingly, in our dataset hands were significantly more frequently examined on the right side; however, we have no explanation for this finding.

Due to the high rate of missing values for laterality in the overall evaluation, a new data analysis was performed, which collected the values for laterality for the body regions where a lateral orientation should actually be present: hands, knees, feet, ankles, and shoulders. However, here too it was shown that most series did not contain a value for laterality, even if the missing rate is lower than in the overall evaluation. The distribution of the series among the values for laterality is shown in Table 1 , with the series with values “B” and “U” being excluded due to the percentages being too small. Series examining knees exhibited the highest rate of available values for laterality. Nevertheless, the percentage of missing values for laterality in these examinations still varied between 46 and 88%.

**Table 1:** The distribution of values for the DICOM tag “Laterality” (0020,0060) in analyzed series of paired body regions

Body region	Percentage of series with missing value for laterality	Percentage of series with laterality value “L”	Percentage of series with laterality value “R”
Hands	88	3	9
Knees	46	29	25
Feet	78	9	13
Ankles	70	15	15
Shoulders	60	20	20

## Discussion

We here developed an end-to-end data processing pipeline that transforms DICOM imaging metadata directly from the clinical routine PACS of the UHE into HL7-FHIR “ImagingStudy” resources. Subsequently, the pipeline ensures a pseudonymization of the resources and finally stores them in an FHIR server for research purposes. Additionally, we provided an analysis tool based on these FHIR resources using state-of-the-art technology, such as Pathling 17 and Apache Spark, 18 providing new insights into imaging studies acquired in two departments of UHE. Because of its reliance on the standardized FHIR format, this analysis tool can be shared with other German DIC, supporting them to perform similar data quality and completeness checks as soon, as they also integrate DICOM image metadata into their FHIR servers. The newly created “ImagingStudy” resources expand the existing research data at our DIC and illustrate a first step toward an integrated view on clinical and imaging data.

### Is the DICOM Standard Currently Sufficient?

The DICOM standard plays a pivotal role in extracting information from PACS, significantly enhancing access to medical images and their metadata, facilitating interoperability across various systems. While compliance with DICOM is voluntary, it's crucial for interoperability between imaging systems and PACS. For example, DICOM allows manufacturers to include proprietary data, so-called “private tags,” into the DICOM header, which on the one hand enables great flexibility, but on the other hand also poses challenges, for example, with regard to interoperability and data harmonization as the DICOM standard does not regulate the meaning and encoding of those private tags. 22 Inconsistencies in implementation have led to interoperability issues caused by the standard's complexity and the absence of validation mechanisms. To address these challenges, Larobina proposed to develop software tools that can verify compliance with the standard. 22

In our analysis, we detected some weaknesses of the DICOM metadata, which led to incomplete or inaccurate entries. Such problems were also highlighted before in other studies. 23 Especially, the specification of the body region examined seems to be challenging in clinical workflows: predefined examination protocols are often selected by staff, implying specific imaging parameters for particular body parts. However, due to patient anatomical variations, protocols for other body areas than the examined region could be used to achieve better image quality, which can lead to inaccurate entries in the DICOM tag “ *Body Part Examined* .” 23 The study of Gueld et al found that typical mislabelings are “ABDOMEN” instead of “CHEST” and “HEAD” instead of “CHEST.” 23 Some of the here observed missing values regarding the DICOM tag “ *Laterality* ” could potentially be related to this phenomenon, although we did not find systematic issues by random checks. We further found that the list of available body regions in the data with which we tested the pipeline is significantly smaller than the dataset actually provided by DICOM for this purpose, which consists of more than 300 body regions. 2 Potential solutions primarily lie with manufacturers, who could adjust the mapping of examined body regions to adhere more closely to the DICOM standard. Also, consideration could be given, however, to whether the subdivision into over 300 defined body regions in the DICOM standard is really necessary and practical, given the level of granularity achievable in reality.

Moreover, when new versions of the DICOM standard are released, the expected or permitted values for the DICOM tags may change. For example, the value “LEG” was available up to version 2022d of the DICOM standard in “ *Body Part Examined* ,” whereas in later versions, this was replaced with “LOWERLEG.” 19 We here used the SNOMED-CT mapping for body part examined present in the current DICOM version (version 2024a) and applied it to imaging studies from 2022. However, to avoid missing or wrong mappings, it would be necessary to always use the SNOMED-CT mapping for FHIR generation valid in the respective year in which the DICOM study was generated. In the future, our pipeline could be enhanced to take this into account. For this to work reliably, however, it is also necessary for the respective device manufacturers to ensure that the DICOM tags always remain in conformity with the respective valid DICOM version.

Proposed solutions for the discrepancies between DICOM and SNOMED-CT include standardizing mappings and exploring alternative DICOM tags like “ *Anatomic Region Sequence* ” 24 25 to enhance interoperability and provide detailed region descriptions. Other studies tried automated detection of body regions using machine learning models. 26 27 A further option to enhance the quality and completeness of the image metadata concerning the examined body region in the FHIR research repositories could involve extracting these data element from the hospital's Radiological Information System (RIS) instead of relying solely on the DICOM image header. The RIS is a dedicated system used in radiology departments to manage and store structured information about imaging orders, procedures, and associated administrative data. It typically contains detailed, manually entered information on the clinical context of an imaging procedure, including the specific body region examined. If the RIS data are more complete and of better quality, however, still needs to be verified.

Regardless of which solution is ultimately chosen in the future, although having analyzed just a small subset of available attributes, our results also demonstrate that while the DICOM standard is widely used, it was primarily designed with clinical care documentation in mind, not necessarily with a focus on providing DICOM metadata for research purposes. However, if reliable queries and algorithms are to be developed to analyze DICOM metadata or select image studies based on their metadata, a method must be devised to reliably capture the examined body region and other information in the clinical documentation.

### A Suitable FHIR Resource Type

The current specification of the “ImagingStudy” resource type in FHIR presents limitations in capturing metadata for research purposes, primarily because many imaging-related information are available at the level of DICOM series, instead of the DICOM study, which would be necessary for meaningful feasibility queries. Otherwise, it would become challenging to provide aggregated results for specific queries based on study-level data. This could be solved by, e.g., expanding the “ImagingStudy” resource type and summarizing important information from the series level on the study level. Furthermore, it needs to be considered that currently only a small subset of available DICOM attributes is included in the definition of the “ImagingStudy” FHIR resource type. To provide extensive and useful metadata for research purposes, effort is required to add much more DICOM attributes to the resource type, preferably also taking modality-specific attributes into account. Before starting such an endeavor, however, the completeness and quality of such attributes should be verified again, to prevent modelling attributes in the “ImagingStudy” FHIR resource type for which in the real world rarely data are documented.

To address this issue within the German MII and to follow its goal of continuously evolving its core dataset (CDS), a team was formed in 2021 to develop an “Imaging Procedures” extension module, intended to be integrated into the CDS. Efforts are directed toward two main perspectives: capturing structured radiological reports and mapping DICOM header metadata into the FHIR format. The latter aspect, informed by insights from this study, includes to introduce the mapping of examined body regions at the study level and to integrate additional DICOM attributes, especially modality-specific attributes such as the radiation dose in X-ray-based imaging systems. This is going to enhance the granularity for future feasibility queries, providing researchers with a comprehensive set of attributes relevant to their investigations. Once finalized, the MII's CDS extension module will replace the currently used HL7-default “ImagingStudy” (R4) resource type for DICOM-to-FHIR conversions at UHE. While certainly adjustments to the DICOM-to-FHIR converter are required, the modular design of the pipeline enables straightforward adaptations.

### Optimizing the DICOM-to-FHIR Pipeline

Although the DICOM-to-FHIR pipeline developed in this project achieved a satisfactory rate of more than four DICOM studies per minute, there is still room for improving its processing speed and overall efficiency.

To enhance the efficiency of the pipeline, extracting only the DICOM headers without pixel data could be beneficial. However, the currently used PACS system at UHE does not support this feature, necessitating the transfer of all DICOM studies including image data, which restricts performance.

Another issue lies in the pipeline structure, as some DICOM studies could not be transferred completely. This is due to non-image objects of certain SOP classes that cannot be processed by the CTP DICOM endpoint. A potential solution could include to define a whitelist of SOP classes, requiring extensive research and collaboration with the local PACS team.

In the long term, DICOM metadata could be stored in a dedicated database to improve efficiency by storing DICOM metadata independently from image data and thus allowing faster and more flexible access for research purposes. This “header database” should be a dedicated database system designed to extract, store, and manage the metadata contained in DICOM headers, such as patient information, imaging parameters, and study details, without requiring access to the pixel information of the images. Suitable database management systems for this purpose include, for example, MongoDB, PostgreSQL, or ElasticSearch, and a proof-of-concept implementation could help to identify the most effective solution for managing and querying large-scale imaging metadata in a research context.

### Limitations

The major limitation of this project is that the here presented DICOM-to-FHIR pipeline and the data quality/completeness analysis performed have been implemented and pursued only at one German university hospital. Thus, our results might not be completely transferable to other sites and may not be representative for the overall imaging data landscape in Germany. However, to transfer and deploy this pipeline also to other university hospitals as well as pursuing the same data quality/completeness analysis at further MII DICs would be a worthwhile effort toward a large-scale integration of clinical and imaging data throughout Germany.

### Possible Applications of the DICOM-to-FHIR Pipeline

Our DICOM-to-FHIR pipeline offers versatile applications in medical research, notably in feasibility assessments and federated analyzes. Integrating DICOM data into FHIR streamlines access to structured clinical data, enhancing researchers' ability to carry out feasibility queries efficiently.

One possible application is the integration into platforms like the German Portal for Medical Research Data (FDPG) 28 29 or the Bavarian Oncology Real World Data Research Platform. 30 By incorporating DICOM metadata, researchers can conduct comprehensive feasibility queries and cohort selections, now also leveraging basic imaging-specific information such as modality and location. This improves analysis precision and facilitates suitable cohort identification.

Another field of application for the generated FHIR resources are federated analyzes, enabling the combination and analysis of data from multiple sites while ensuring data privacy. Leveraging federated approaches in image-based studies can enhance existing infrastructures like the Radiological Cooperative Network (RACOON) 31 and the Bavarian Oncology Radiology Network (BORN), 32 broadening project capabilities.

The pipeline also lays the foundation for Radiomics, the extraction of high-dimensional data from medical images for comprehensive analysis. By converting DICOM metadata into FHIR, the pipeline facilitates streamlined access to imaging data, empowering researchers to develop precision medicine approaches, particularly in oncology, 33 by identifying patient cohorts based on clinical and imaging features. 34

## Conclusion

The DICOM-to-FHIR pipeline developed in this thesis lays the groundwork for leveraging DICOM image metadata in various research contexts. Insights gained from analyzing DICOM studies highlight challenges in standardization within the DICOM standard itself, pointing to the need for both industry adjustments and innovative solutions like artificial intelligence-assisted metadata detection. 26

While further refinement is necessary, the pipeline's generated resources in FHIR format can already support feasibility assessments e.g., within the MII and its Portal for Medical Research Data, 28 29 facilitating future research characterizations and cohort definitions. Integration with other clinical parameters promises a comprehensive understanding of disease progression and treatment effects, enriching the research landscape and enabling the development of new diagnostic and therapeutic approaches.
